# Intrauterine hyperglycemia impairs memory across two generations

**DOI:** 10.1038/s41398-021-01565-7

**Published:** 2021-08-20

**Authors:** Kexin Zou, Jun Ren, Sisi Luo, Junyu Zhang, Chengliang Zhou, Chengxi Tan, Pingping Lv, Xiao Sun, Jianzhong Sheng, Xinmei Liu, Hefeng Huang, Guolian Ding

**Affiliations:** 1 The International Peace Maternity and Child Health Hospital, School of Medicine, Shanghai Jiao Tong University, Shanghai, China; 2 Shanghai Key Laboratory of Embryo Original Diseases, Shanghai, China; 3 Research Units of Embryo Original Diseases, Chinese Academy of Medical Sciences, Shanghai, China; 4 Key Laboratory of Reproductive Genetics (Ministry of Education), Department of Reproductive Endocrinology, Women’s Hospital, Zhejiang University School of Medicine, Hangzhou, China; 5 Obstetrics and Gynecology Hospital, Institute of Reproduction and Development, Fudan University, Shanghai, China

**Keywords:** Hippocampus, Comparative genomics

## Abstract

Studies on humans and animals suggest associations between gestational diabetes mellitus (GDM) with increased susceptibility to develop neurological disorders in offspring. However, the molecular mechanisms underpinning the intergenerational effects remain unclear. Using a mouse model of diabetes during pregnancy, we found that intrauterine hyperglycemia exposure resulted in memory impairment in both the first filial (F1) males and the second filial (F2) males from the F1 male offspring. Transcriptome profiling of F1 and F2 hippocampi revealed that differentially expressed genes (DEGs) were enriched in neurodevelopment and synaptic plasticity. The reduced representation bisulfite sequencing (RRBS) of sperm in F1 adult males showed that the intrauterine hyperglycemia exposure caused altered methylated modification of F1 sperm, which is a potential epigenetic mechanism for the intergenerational neurocognitive effects of GDM.

## Introduction

Accumulating evidence suggests that an adverse in utero environment can increase the risk of chronic diseases in later life. Long-term postnatal health may be affected by metabolic experience in utero [1, 2]. Intrauterine hyperglycemia is a major characteristic of gestational diabetes mellitus (GDM) and is associated with a high risk of diabetes in offspring [3]. In humans, it is noteworthy that in addition to metabolic dysfunction, studies have documented that children of mothers with diabetes during pregnancy is associated with the impaired cognitive ability [4–7], although one study failed to detect such association in another population [8]. It is also controversial whether the association between maternal diabetes in pregnancy and offspring cognitive outcomes can be fully explained by shared familial environmental factors or by an intrauterine biological mechanism [9].

Experimental investigations in animals indicated that uncontrolled diabetes mellitus was associated with morphological and functional alterations in the brain [10–12]. Hippocampus, a structure critical to cognitive processes, has been shown to undergo apoptotic cell death when subjected to hyperglycemic insult [13–15]. Diabetes during pregnancy strongly influences the regulation of both insulin-like growth factor-1 receptor (IGF-1R) and insulin receptor (InsR) in the rat hippocampus [13]. Maternal diabetes mellitus can also reduce the expression of synaptophysin (SYP) in the developing hippocampus and cerebellar cortex of neonatal rats [16, 17]. However, the intergenerational effect on the F2 offspring and the underlying molecular mechanism are unclear.

Epigenetic alterations regulate tissue-specific gene expression during growth and development without altering the DNA sequence. DNA methylation primarily occurs on CpG dinucleotides and is generally associated with gene repression when positioned on the promoter region [2, 18]. Our previous research has demonstrated that intrauterine hyperglycemia changed DNA methylation levels on the imprinted gene Igf2/H19 in the F1 pancreatic islet, which was further transmitted to F2 through F1 germ cells [19]. Therefore, we hypothesize that the hyperglycemic intrauterine environment of GDM could result in a high risk of cognitive impairment in F2 offspring by affecting the development or function of the hippocampus through altering DNA methylation in F1 germ cells. We focused on the male offspring in this study because our previous GDM mouse model of intrauterine hyperglycemia indicates that male offspring was more susceptible to such intergenerational effects than females.

## Materials and methods

### Mice

All animal protocols were reviewed and approved by the Zhejiang University Animal Care and Use Committee. At the age of 8 weeks, virgin female ICR mice (*n* = 60) were mated with normal males. The onset of pregnancy was determined by the presence of a copulation plug after overnight mating (designated as day 0 [D0] of pregnancy). After a 12-h fast, the females were randomly divided into a control group and an intrauterine hyperglycemia group with GDM (GDM group). Mice in the GDM group were injected with a single intraperitoneal injection of streptozotocin (STZ; Sigma, St. Louis, MO) in 0.1 mmol/L citrate buffer (pH 4.5) at a dose of 150 mg/kg body wt. Control pregnant females received an equal volume of citrate buffer. Diabetes was confirmed by the measurement of blood glucose concentration via the tail vein as previously described [19]. The pregnant mice were allowed to deliver spontaneously. The litter size was randomly reduced to 10 at birth to assure uniformity. The pups from the GDM group were fostered by normoglycemic females until they were weaned at the age of 3 weeks. Cross fostering was performed after birth immediately. The F1 adults of control (F1-C) female and F1-C male were intercrossed to obtain F2 offspring of the control (F2-C) group. The F1-C female and F1 adults of GDM (F1-GDM) male mice were intercrossed to obtain F2 offspring of the GDM (F2-GDM) group.

### Behavioral tests

All the behavior tests were done from 3-month-old during 12 p.m.–6 p.m. in a dim environment, unless specifically mentioned. Experimenters were blind to experimental group allocation. Mice were given a 1-week interval to recover from the first behavioral test to have a next behavioral test. (1) The open field (OF) test consisted of a clear dark-colored polyvinyl plastic-wall arena measuring 50 cm × 50 cm with 40 cm high walls and a dark floor. The center of 20 cm × 20 cm was demarcated on a computer, leaving a surrounding outer zone of 30 cm width. The behavior of each mouse was recorded with Noldus tracking software. (2) The Y-maze tested spontaneous spatial recognition as a hippocampus-dependent memory test. The Y-maze, a horizontal maze consisting of three arms (40 cm × 3 cm × 12 cm), has arms symmetrically disposed at a 120° angle. The floor and walls of the maze were made with a dark-colored opaque polyvinyl plastic. Mice were placed in one arm. The sequence (e.g., ABCAB) and a number of arm entry were manually recorded for each mouse for an 8-min period. Entry into all three arms on consecutive choices was defined as an actual alteration (i.e., ABC, CAB, or BCA, but not BAB). Between tests, maze arms were cleaned to remove residual odors. The alternation percentage was defined as the following equation: % alternation = [(number of alternations)/(total arm entries – 2)] × 100. Percentage arm alteration is an established behavioral assay for short-term spatial memory. (3) In the novel subject of recognition (novel object recognition [NOR]) test, a mouse is presented with two similar objects in the OF during the familiarization session for 8 min, and then one of the two objects was replaced by a novel object during a second session in which mouse can behave freely for 8 min. NOR discrimination index (DI) was calculated by the following formula: (Time exploring novel object – Time exploring familiar object)/Total object exploration time. The amount of time taken to explore the new object provides an index of recognition memory. Higher DI indicates better recognition memory. (4) The object-in-place task (OiPT) was comprised of an acquisition phase and a test phase separated by a 30 min delay. In the acquisition phase, the subjects were presented with four different objects (A–D). These objects were placed in the corners of the arena 10 cm from the walls. Each mouse was placed in the center of the arena and allowed to explore the objects for 10 min. During the delay period, all the objects were cleaned with alcohol to remove olfactory cues and any sawdust that had stuck to the object. In the test phase, two of the objects, e.g., B and D (which were both on the left or right of the arena), exchanged positions and the mouse was allowed to explore the objects for 10 min. The time spent exploring the two objects that had changed position was calculated. The objects moved (i.e., those on the left or right) and the position of the objects in the sample phase were counterbalanced between mice.

### RNA isolation and quantitative real time‑PCR (qPCR) analysis

Total RNA was isolated from the mouse hippocampus using RNeasy (Qiagen, Valencia, CA). The cDNA was synthesized using oligo-dT and random primers (TaKaRa, Dalian, China) for qPCR (ABI Prism 7900HT; Applied Biosystems, Foster City, CA) with commercial primers generated for the system. Relative expression was calculated using the 2 − ΔΔCq method. GAPDH was used as the internal control. Primers were listed in Table S1.

### Microarray

Microarray was performed in the hippocampus from 4-month-old F1 and F2 offspring. The raw microarray data GSE147039 is available at the NCBI Gene Expression Omnibus (GEO) database (http://www.ncbi.nlm.nih.gov/geo/). The probe set IDs were converted into the corresponding gene symbol using the annotation information derived from platform GPL6887. If multiple probesets correspond to one gene, the mean expression values of those probesets were obtained. The limma package V3.34.9 in R was used to identify the differentially expressed genes (DEGs) in F1 and F2 hippocampus compared with the control group [20]. The DEGs were screened out according to adjusted *P* value < 0.05 and | log2FoldChange | > 1. The clusterProfiler V3.8.0 in R was used to identify and visualize the Gene ontology (GO) terms and Kyoto Encyclopedia of Genes and Genomes (KEGG) pathways enriched by DEGs [21]. *P* value < 0.05 was considered as a significant enrichment. GSEA of KEGG gene sets was run using 1000 gene_set permutations by clusterProfiler. The normalized enrichment score (NES) was regarded as the primary statistic for examining GSEA enrichment results.

### Reduced representation bisulfite sequencing

Reduced representation bisulfite sequencing (RRBS) was performed in sperm obtained from the caudal epididymis of 4-month-old F1 male mice (Genergy Biotechnology Co., Ltd., Shanghai, China). Briefly, 5 μg genomic DNA was digested using the methylation-insensitive restriction enzyme MspI (New England Biolabs, Beverly, MA, USA). A Qiagen Mini Purification kit (Qiagen, Hilden, Germany) was used to purify the digested products. Then, the ends of each restriction fragment were filled in and adenosine was added at the 3′-end. Methylated paired-end Illumina adapters were ligated to the ends of the DNA fragments using T4 DNA ligase, and fragments sized 100–200 bp were purified by agarose gel extraction. The purified fragments were treated with sodium bisulfite and then amplified by PCR. The final PCR products were sequenced on HiSeq 2500 (Illumina Inc., San Diego, CA, USA). Differentially methylated loci (DML) and differentially methylated regions (DMRs) were analyzed based on a Bayesian approach [22], summarized as follows as our previous study:[23] two groups were modeled according to the Bayesian stratification model, and the Wald test was applied to each locus to get a *p* value for each CpG site. For each CpG site, a difference in methylation value between two groups ≥5% and a posteriori probability of Wald test ≥0.95 was considered to be a DML. A methylation region was defined as a DMR when it met these three criteria: (1) the length of this region was at least 50 bp; (2) the region contained no less than three CpG sites; (3) the proportion of DMLs in this region was no less than 50%. When a DMR showed no less than 50% overlap with one element of the gene, it was defined as a differentially methylated gene (DMG). RRBS reads were mapped to the reference mouse genome (mm10) by Bismark (version 0.16.3). DSS V2.30.1 in R was used to detect DMRs. The RRBS data reported in this paper have been deposited in the GEO database with accession number GSE142502.

### Statistical analysis

Data were shown as the mean ± SEM. A priori sample size calculation was not performed, but our sample sizes are similar to those reported in previous publications. Shapiro–Wilk (*n* < 10) and D’Agostino and Pearson omnibus (*n* > 10) normality tests were performed to determine if values fit a Gaussian distribution. For all behavioral studies, a two-tailed unpaired Student’s *t*-test was used to analyze the significance between groups. The *t*-test and Benjamini–Hochberg method were used to calculate the *P* value and FDR of microarray, respectively. GO analysis was performed using a hypergeometric distribution test. All the statistical analyses were conducted with GraphPad Prism 7 (GraphPad Software, Inc) and R (version 3.6.2). Differences were considered statistically significant at *p* < 0.05.

## Results

### Intrauterine hyperglycemia affects cognition of both F1 and F2 male offspring

**Fig. 1:**
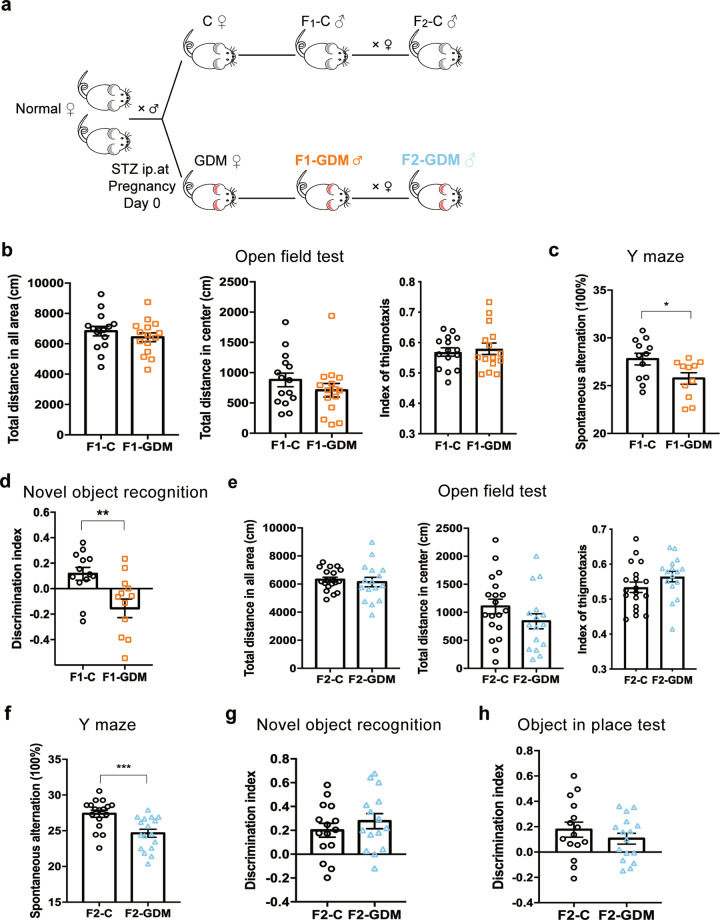
Intrauterine hyperglycemia impairs spatial memory in F1 offspring. **a** Experimental design. **b** Open field test of F1 offspring of control (F1-C) and GDM (F1-GDM) mice (*n*_F1-C_ = 15, *n*_F1-GDM_ = 15 male 3-month-old mice). **c** Y maze test of F1-C and F1-GDM mice (*n*_F1-C_ = 12, *n*_F1-GDM_ = 11 male 3-month-old mice). **d** Novel object recognition test of F1-C and F1-GDM mice (*n*_F1-C_ = 13, *n*_F1-GDM_ = 13 male 3-month-old mice). **e** Open field test of F2 offspring of control (F2-C) and GDM (F2-GDM) mice (*n*_F2-C_ = 19, *n*_F2-GDM_ = 16 male 3-month-old mice). **f** Y maze test of F2-C and F2-GDM mice (*n*_F2-C_ = 17, *n*_F2-GDM_ = 17 male 3-month-old mice). **g** Novel object recognition test of F2-C and F2-GDM mice (*n*_F2-C_ = 15, *n*_F2-GDM _= 15 male 3-month-old mice). **h** Object in place test of F2-C and F2-GDM mice (*n*_F2-C_ = 15, *n*_F2-GDM_ = 15 male 3-month-old mice). Data were analyzed by a two-tailed unpaired *t*-test from three independent experiments. *P < 0.05 vs. F1-C; **P < 0.01 vs. F1-C; ***P < 0.001 vs. F2-C.

We induced moderate hyperglycemia during pregnancy through injection of STZ. Male F1 adults were then intercrossed to unexposed females to obtain F2 offspring (Fig. 1a). Both F1 and F2 offspring were analyzed in behavioral tests at 3–4 months old.

The anxiety levels and locomotor activity was assessed in the OF test. Compared with the F1-Control group, F1-GDM mice showed normal total explorative distance, total explorative activity in the central area, and the index of thigmotaxis (Fig. 1b). Based on the normal explorative activity, we further investigate the spatial memory ability between F1-control and F1-GDM. F1-GDM mice showed less spontaneous alteration than F1-control in Y-maze (Fig. 1c), suggesting impaired spatial memory. A similar result was observed in the NOR test. In the NOR test, the amount of time spent with the novel object compared with the total time spent exploring both objects represents an index of recognition memory. Compared to F1-control, F1-GDM mice spent less time investigating the novel object despite similar total exploration times, revealing a remarkable memory deficit (Fig. 1d).

Similar to F1 mice, F2-GDM mice showed normal locomotor activity and did not have an anxiety disorder (Fig. 1e), but displayed a significant deficit in spatial memory in the Y-maze test although there was no obvious difference in NOR test or OiPT associative recognition memory (Fig. 1f–h). These results suggest that intrauterine hyperglycemia impairs spatial memory in both F1 and F2 male offspring.

### Intrauterine hyperglycemia disrupts hippocampal transcriptome in F1 offspring

To explore the long-term effects of intrauterine hyperglycemia on transcriptional reprogramming, we used microarray to analyze the transcriptome of hippocampi from 4-month-old F1 (*n* = 5 per group). Using a stringent threshold of adjusted *P* value < 0.05 and | log2FoldChange | > 1, we identified a total of 451 DEGs, including 218 upregulated and 234 downregulated genes, in F1-GDM compared to control. (Table S2).

**Fig. 2:**
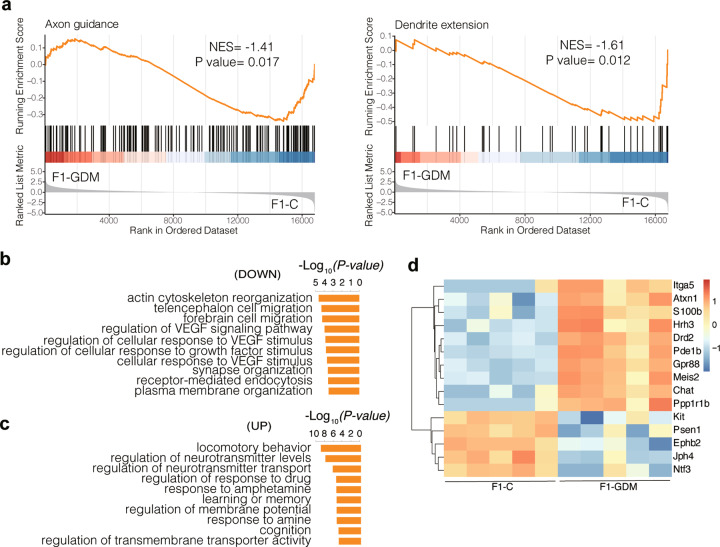
Intrauterine hyperglycemia disrupts hippocampal transcriptome in F1 offspring. **a** GSEA for male progenies’ comparison (*n* = 5 per group). Graphs for axon guidance, dendrite extension, and their respective *P* value and normalized enrichment score (NES) are shown. **b**, **c** Top ten GO terms enriched in down- and up-regulated DEGs between F1-GDM and control group. **d** Heatmap of up- or down-regulated DEGs detected in F1 hippocampus that enriched to cognition (GO:0050890). NES absolute value of normalized enrichment score, VEGF vascular endothelial growth factor.

GSEA of the hippocampi transcriptome in F1-GDM vs. Control revealed the robust enrichment of curated gene sets for axon guidance (KEGG:mmu04360) and dendrite extension (GO:0097484), indicating that the expression of member genes were decreased in the F1-GDM group (NES < −1, *P* value <0.05) (Fig. 2a and Table S3). Axon guidance is a process by which axons stretch to their correct targets and plays a key role in building neuronal circuitry [24]. Dendrite growth and synapse formation occur concurrently during development, these processes may be coordinated and interdependent [25]. GO analysis revealed that down- and up-regulated DEGs were mainly enriched in “forebrain cell migration”, “synapse organization” and “cognition” (Fig. 2b, c). Heatmap shows the genes related to cognition (GO:0050890) were significantly up- or down-regulated in F1 offspring (Fig. 2d). Among them, mice overexpressing S100b show enhanced excitotoxicity, altered synaptic plasticity, and cognitive impairment [26]. Hrh3 encodes the histamine receptor H3, which is ubiquitously released from neurons and can regulate neurotransmitter release [27]. Researchers also reported that Drd2 has a role in neuronal maturation and dopaminergic synapse formation [28]. Gpr88 is implicated in a large repertoire of behavioral responses including spatial learning [29]. Meis2 is associated with impairments in working memory and cognition [30]. Neurotrophin-3 (Ntf3) belongs to the family of highly conserved dimeric growth factors that functions as a positive modulator of synaptogenesis involving TrkC and PTPσ [31]. In summary, intrauterine hyperglycemia altered gene expression patterns in the hippocampus of the adult F1 male offspring, and the DEGs were highly enriched in neural development and synapse function.

### Epigenetic changes in the F1 germ cells

**Fig. 3:**
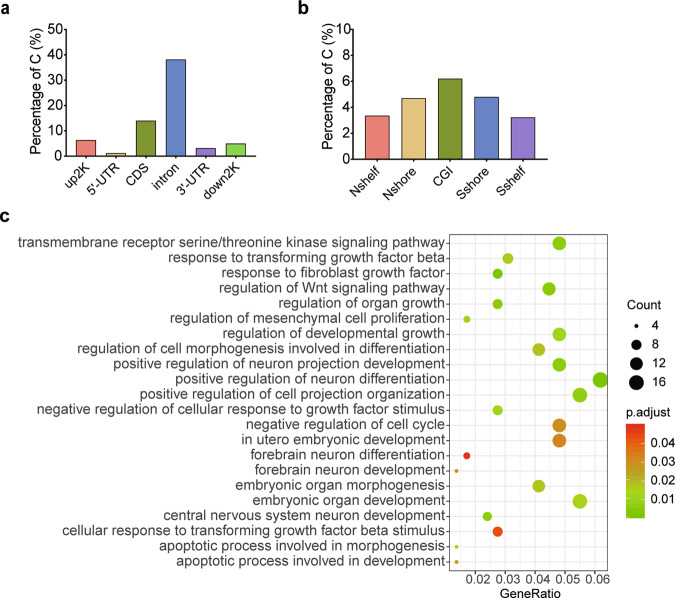
Epigenetic changes in the F1 germ cells. **a** Distribution of differentially methylated loci in F1-GDM sperm. **b** Distribution of differentially methylated loci in CGI and neighboring regions. **c** GO analysis of differentially methylated genes in F1-GDM sperm.

Since the F1-GDM male were directly exposed to hyperglycemia in utero, their cognitive impairment could be due to direct disruption of neurodevelopment by hyperglycemia rather than any epigenetic changes. Whether cognition impairment and gene expression changes in F2-GDM is more likely caused by epigenetic mechanisms, such as DNA methylation. We performed RRBS to search for DML in F1-GDM sperms vs. F1-control. Intrauterine hyperglycemia exposure resulted in 64,658 DML that were distributed in the upstream 2 k (6.36%), 5′-untranslated region (5′-UTR, 1.24%), coding sequence (CDS, 14.01%), introns (38.18%), 3′-UTR (3.17%), downstream 2 k (4.99%), and other elements (38.75%) of genes (Fig. 3a). We also investigated the distribution across CpG islands (CGIs) and neighboring regions (Fig. 3b). CpG island shore was defined as 2 kb regions flanking a CpG island, and CpG shelf as a 2 kb region outside a CpG shore (away from the CGI). GO analysis identified a cluster of DMGs that were strongly related to “neuron development”, “neuron differentiation” and “organ growth” (Fig. 3c).

### Intrauterine hyperglycemia disrupts hippocampal transcriptome in F2 offspring

To explore the intergenerational effects of intrauterine hyperglycemia on transcriptional reprogramming, we used microarray to analyze the transcriptome of hippocampi from 4-month-old F2 (*n* = 5 per group). Using a stringent threshold of adjusted *P* value <0.05 and | log2FoldChange | >1, we identified a total of 1050 DEGs, including 511 upregulated and 539 downregulated genes, in F2-GDM vs. control (Table S4).

**Fig. 4:**
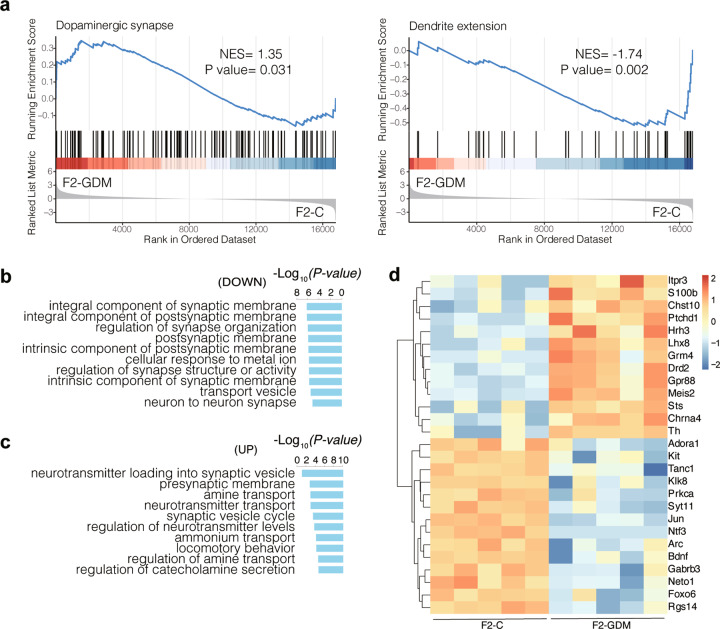
Intrauterine hyperglycemia disrupts hippocampal transcriptome in F2 offspring. **a** Enrichment plots and statistical analysis from GSEA. **b**, **c** Top ten GO terms enriched in down- and up-regulated DEGs between F2-GDM and control group. **d** Heatmap of up- or down-regulated DEGs detected in F2 hippocampus that enriched to cognition (GO:0050890). NES absolute value of normalized enrichment score.

GSEA of preranked genes in F2 offspring also indicated significant inhibition in dendrite extension (GO:0097484) (NES <−1, *P* value <0.05). GSEA also provided insights into changes of activated pathways in F2-GDM, including dopaminergic synapse (KEGG:mmu04728) pathway (NES >1, *P* value <0.05) (Fig. 4a and Table S5). GO analysis revealed that down- and up-regulated DEGs were also mainly enriched in “synapse organization”, “postsynaptic membrane” and “neuron to neuron synapse”, including several shared GO terms in both F1 and F2 hippocampus (Fig. 4b, c). Heatmap shows the genes related to cognition (GO:0050890) were significantly up- or down-regulated in F2 offspring (Fig. 4d). Although the overall change of gene sets including the axon guidance (KEGG:mmu04360) and neuroactive ligand-receptor interaction (KEGG:mmu04080) in the F2 generation was moderate compared to the F1 generation (Tables S3 and S5), the F2 generation showed more DEGs related to cognition (GO:0050890), such as brain-derived neurotrophic factor (BDNF), compared to the F1 generation (heatmap in Figs. 2d, 4d). Literature has reported that hippocampus degeneration with diminished BDNF leads to a decline in cognition [32]. In addition, we performed GSEA by microarray data to compare the F2-GDM versus F1-GDM and confirmed a statistically significant enrichment of insulin resistance (KEGG:mmu04931) gene set (Supplementary Fig. 1), which could contribute to the impaired working memory function. In summary, intrauterine hyperglycemia altered gene expression patterns in the hippocampus of the adult F2 male offspring as well, and the DEGs were highly enriched in neural development and synapse function.

### Overlapping DMGs in F1 sperm and differentially expressed genes in F2 hippocampus

By overlapping DMGs of sperm in F1 offspring and expressed genes of the hippocampus in F2 offspring, we found 56 genes hypermethylated in F1-GDM sperm compared to Control and the tendency of 56 gene expression are downregulated (logFC <0) in F2-GDM hippocampus compared to Control (Supplementary Fig. 2). GO analysis showed that 56 genes were enriched in “neuron to neuron synapse”, “postsynaptic density/specialization”, “neuron projection development”, “structural constituent of synapse/postsynapse”, including Akap7, Camk2b, Dlgap1, Tanc1, Tubb2b, Wnt5a, and Zeb2, most of which were downregulated (logFC <0) in the hippocampus of both F1-GDM and F2-GDM. Additionally, within the nerve system development (GO:0007399) term in MGI database (http://www.informatics.jax.org/), a network of hypermethylated genes related to axon guidance (Htr7), positive regulation of astrocyte differentiation (Fryl), negative regulation of oligodendrocyte differentiation (Nfix), regulation of postsynaptic density assembly (Hoxb3), had a tendency for low expression in the F2-GDM versus the Control samples (Table 1). Of these genes, hypermethylated CpGs was most frequent in CDS regions, followed closely by intron regions.

**Table 1:** The crucial genes associated with central nervous system development are hypermethylated in F1-GDM sperm and have a tendency to be downregulated (logFC <0) in the F2-GDM hippocampus.

Symbol	RRBS of Sperm (F1-GDM vs. Ctrl)					Microarray of Hip (F2-GDM vs. Ctrl)	
	Start	End	mean Methy Ctrl	mean Methy F1-GDM	Element	logFC	adj. *P* value
Tubb2b	34127486	34127641	16.03%	81.88%	CDS	−3.0865	0.001
Htr7	35969594	35969756	7.22%	73.70%	CDS 3′-UTR	−1.7638	0.249
Dlgap1	70516618	70516771	6.80%	86.87%	CDS	−1.2927	0.186
Tanc1	59843298	59843455	65.81%	81.41%	CDS	−1.1135	0.026
Zeb2	44988693	44988893	14.49%	85.39%	CDS	−0.7887	0.645
Wnt5a	28518390	28522909	26.62%	76.36%	CDS Intron	−0.5713	0.211
Hoxb3	96345925	96346054	7.08%	28.65%	CDS	−0.4266	0.644
Nfix	84784452	84784657	27.85%	45.75%	Intron	−0.2088	0.678
Nfix	84704134	84704384	38.83%	64.20%	Intron	−0.2088	0.678
Akap7	25251588	25251727	25.82%	77.69%	Intron	−0.2014	0.529
Camk2b	6010025	6010230	49.49%	89.20%	Intron	−0.1977	0.253

*RRBS* reduced representation bisulfite sequencing, *Hip* hippocampus, *F1-GDM* the first filial of gestational diabetes mellitus, *F2-GDM* the second filial of gestational diabetes mellitus, *Ctrl* control group.

### Common transcriptomic signatures across two generations

**Fig. 5:**
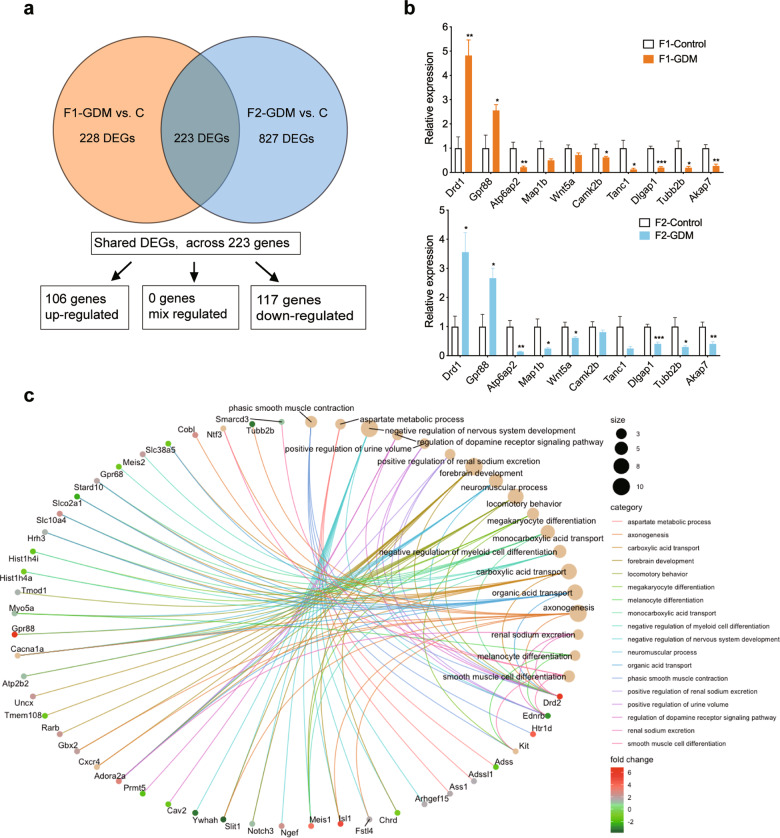
Common transcriptomic signatures across two generations. **a** Venn diagram of differential genes overlapped between F1-GDM vs. control and F2-GDM vs. control. **b** The expression of meaningful shared DEGs in F1 and F2 offspring (*n*_F1-Control_ = 4, *n*_F1-GDM_ = 5, *n*_F2-Control_ = 4, *n*_F2-GDM_ = 5 male 4-month-old mice). **c** Enrichment analysis of shared DEGs in F1 and F2 offspring. Data were analyzed by a two-tailed unpaired *t*-test. *P < 0.05 vs. control; **P < 0.01 vs. control; ***P < 0.001 vs. control.

By overlapping DEGs of the hippocampus in F1 and F2 offspring, we found that there were 106 genes upregulated, and 117 genes downregulated, in both F1-GDM and F2-GDM mice compared to their respective control mice. All genes were changed in the same direction between these two generations (Fig. 5a). Among the overlapped dramatically altered genes of F1-GDM and F2-GDM offspring, the most biologically significant genes were further validated by RT-qPCR analysis, including the genes regulating hippocampal synaptic plasticity and learning (Camk2b and Drd1), regulating the density and activity of glutamate receptors (Dlgap1 and Tanc1), neurogenic Wnt signaling pathways (Wnt5a and Atp6ap2), and some other genes associated with the hippocampal function (Akap7, Tubb2b, Map1b, and Gpr88) (Fig. 5b). Enrichment analysis of 223 shared DEGs indicated that GO terms were mainly involved in the biological process such as “negative regulation of nervous system development”, “regulation of dopamine receptor signaling pathway”, “forebrain development”, “negative regulation of neuron differentiation”, “axon development”, and “regulation of neurotransmitter transport and synapse organization” (Fig. 5c). These results highlights that intrauterine hyperglycemia leads to persistent and consistent transcriptomic changes across two generations, which may affect hippocampal synaptic plasticity and contribute to memory impairment.

## Discussion

The association of GDM with offspring cognitive deficits has been investigated in a number of epidemiological studies [33, 34]. Further, a systematic review and meta-analysis found that according to 19 articles among 18,681 exposed and more than 2.8 million control participants, exposure to maternal preexisting diabetes in pregnancy was not only related to an impaired intelligence ability in the offspring, but also increase the risk of autism spectrum disorders [35]. The data suggest there is a signal that GDM may be associated with adverse neurocognitive and behavioral outcomes past the neonatal period. However, the underlying mechanisms leading to a higher susceptibility of the progeny to develop cognitive abnormalities later in life involve a complex pathophysiological change.

Maternal metabolic disorders could bring about sex-specific changes in the neurodevelopmental process of growing fetus. It is noteworthy that male offspring are at a higher risk of developing neurodevelopmental disorders. Previous studies showed that fetal exposures to the adverse maternal environment are significant risk factors for neuropsychiatric disease predisposition, in particular in male offspring [36–38]. The most frequently used model of type 1 diabetes is the streptozotocin (STZ) model. STZ is a glucosamine-nitrosourea antibiotic that is similar structurally to glucose and is taken up preferentially by the GLUT2 glucose transporter in insulin-producing pancreatic β-cells [39]. Intraperitoneal treatment with STZ results in β-cell toxicity and necrosis, leading ultimately to insulin deficiency [40]. In our previous study, we also used the STZ-induced GDM model to find that the effect of intrauterine hyperglycemia on male offspring was more obvious than female, with parental characteristics and sex-specific transmission [19]. Differential effects of sex hormones may also explain this sex difference. Estrogen is known to promote neurogenesis in the hippocampus, and an alteration in the response of the hippocampus to estrogen may protect intrauterine environment-related changes in the female brain. Therefore, in this study, we focused on the male offspring. The sex difference in intrauterine hyperglycemia transfer to the brain has not been studied, thus further research is required.

In both F1-GDM and F2-GDM male offspring of mice, there was no difference with control in the OF test, suggesting that the change of learning and memory was not confounded by the lack of locomotor activity. As the hippocampus-dependent memory test, the Y-maze showed the spontaneous spatial recognition was significantly decreased in F1-GDM offspring. In the NOR test, F1-GDM mice spent significantly less times investigating the novel object and control, suggesting the ability to recognize new items significantly decreased. It’s interesting that the impaired working memory function was more obvious in the F2-GDM offspring. Although there was no difference in novel object recognition or object-in-place test between F2-GDM and control. All these phenotypes indicated that the intrauterine hyperglycemia exposure could result in impairment of cognition and memory not only in F1 but also in F2 male offspring.

The clinical and basical evidence have suggested that the disturbances in intellectual and behavioral functioning observed in the children of diabetic women are accompanied by modification of hippocampus structure and function. Investigation of the mechanisms responsible for maternal diabetes-related changes in the development of the hippocampus is helping to prevent impaired cognitive and memory functions in offspring [41]. In this study, the DEGs of F1-GDM and F2-GDM offspring were mainly enriched in “learning or memory”, “cognition”, and “neuron to neuron synapse”. By overlapping DEGs of the hippocampus in F1-GDM and F2-GDM offspring, totally we found the same 106 upregulated genes and 117 downregulated genes. It is interesting that there is no differentially expressed gene showing inconsistent tendency in F1-GDM and F2-GDM mice. The function of these DEGs included regulation of nervous system development, neuron differentiation, and axon development.

Beyond the shared genes associated with cognition (GO:0050890) mentioned above in the Result section, some important overlapped DEGs of F1-GDM and F2-GDM offspring were screened and verified in our study. Camk2, the calcium/calmodulin-dependent kinase type II, is the holoenzyme of the forebrain predominantly, which consists of heteromeric complexes of the Camk2a and Camk2b isoforms, regulating hippocampal synaptic plasticity and learning [42, 43]. Dopamine receptor Drd1 agonist could result in Camk2 activation, glutamate receptor exocytosis, synaptic reorganization, and expression of early markers of hippocampal synaptic plasticity [44]. Wnt5a regulates neuronal morphogenesis during embryonic development, and maintains dendritic architecture of pyramidal neurons in the adult hippocampus, through activating Wnt/JNK and Wnt/Camk2 signaling [45, 46]. As a core protein involved in neurogenic Wnt signaling pathways, Atp6ap2 is critical for proliferating adult neural stem cells and differentiating neuroblasts, essential in early brain development, adult hippocampal neurogenesis, and in cognitive functions. Lack of Atp6ap2 leads to cognitive impairment and neurodegeneration, and mutations of Atp6ap2 in humans are associated with intellectual disability [47, 48]. Postsynaptic density proteins (PSD) play a critical role in regulating the density and activity of glutamate receptors. As a scaffold protein localized at the PSD of glutamatergic neurons, Dlgap1 knockout leads to disruption of protein interactions in the PSD, and deficits in sociability [49]. And, TANC1 is a PSD-95-interacting synaptic protein that contains multiple domains for protein–protein interactions, important for dendritic spine maintenance and spatial memory [50]. The protein kinase A anchoring protein Akap7, a member of tubulin genes family Tubb2b, microtubule-associated protein Map1b, and some other genes associated with hippocampal function were also changed in F1-GDM and F2-GDM offspring.

There may be changes in the cellular composition of the hippocampus (for example, changes in the ratio of neurons:glia), and this may be also one of the potential mechanisms. Hippocampus is a complex brain structure embedded deep into the temporal lobe. It has a major role in learning, memory, and spatial navigation. Notably, the hippocampus has a mixed cellular environment with multiple cell types, including neurons (excitatory pyramidal cells, inhibitory interneurons) and glia (microglia, astrocytes, and oligodendrocytes) [51], and both neurons and glia display heterogeneous morphologies. Glia interacts with neuronal synapse can modulate synaptic transmission and plasticity, and both cell types impact learning and memory. As neurons and glia work together in complex, interdependent networks, it is difficult to isolate and disentangle the relative contribution and role of glia in neurological manifestations. Thus, the work described here, which briefly focuses on the transgenerational effect and hippocampal-related cognition, used the whole hippocampus for gene expression analyses as other studies do [52, 53].

The explicit mechanism of maternal effect on offspring is still unclear. A few studies indicated that maternal overnutrition could induce cognitive deficits across several generations [54]. For F1 offspring, as a mediator of stress effects on neurodevelopmental reprogramming, the placenta may play an important role in the transmission of the maternal adverse environment and effects on the developing brain [55]. Dysregulation of imprinted genes is a plausible mechanism linking maternal stressors with fetal growth [56]. For the mechanism of transmission to F2 offspring, based on our previous research that intrauterine exposure alone is sufficient to cause the epigenetic inheritance in F2 offspring [19, 23], we mainly investigated the methylation status of F1 sperm, finding 56 genes downregulated (logFC <0) in F2-GDM hippocampus hypermethylated in F1-GDM sperm. Our result confirmed that the epigenetic memory carried by DNA methylation pattern could be reprogrammed in F1 germ cell during fetal development in the uterus.

In conclusion, in this study, with the mouse model, we firstly investigated the intergenerational effect of intrauterine hyperglycemia on cognition and memory in offspring and the potential molecular mechanism. The results showed that intrauterine hyperglycemia exposure could result in impairment of cognition and memory in both F1 and F2 male offspring. The DEGs in both F1 and F2 hippocampi were mainly enriched in learning or memory, cognition, and other neuron function. Further research found the altered methylated modification of sperm in F1 adult caused by intrauterine hyperglycemia exposure. Therefore, epigenetic alteration may play important role in the intergenerational transmission of GDM-induced abnormal neurodevelopment. It is essential that future studies focus on identifying the potential mechanism of the maternal effect on epigenetic regulation in the fetus and even their germ cells. To study the effect of intrauterine high glucose environment on the hippocampus of offspring mice more comprehensively, we plan to do the transcriptome sequencing and metabolomics in fetal mice hippocampi to better explain the direct effect of intrauterine hyperglycemia on the hippocampal development of offspring mice.

## Supplementary information

Supplementary Fig 1

Supplementary Fig 2

Table S1

Table S2

Table S3

Table S4

Table S5

## Data Availability

No custom code was used for analysis. RRBS and microarray analysis were performed using R function calls in the publicly available Bioconductor R packages. The datasets generated during this study are available at the Gene Expression Omnibus (GEO) Repository: GSE147039 and D GSE142502.
