# Supplementary figures and images for: Intrauterine hyperglycemia impairs memory across two generations
Source: Transl Psychiatry. 2021 Aug 20;11:434. doi: 10.1038/s41398-021-01565-7 (PMC8379206; doi:10.1038/s41398-021-01565-7)

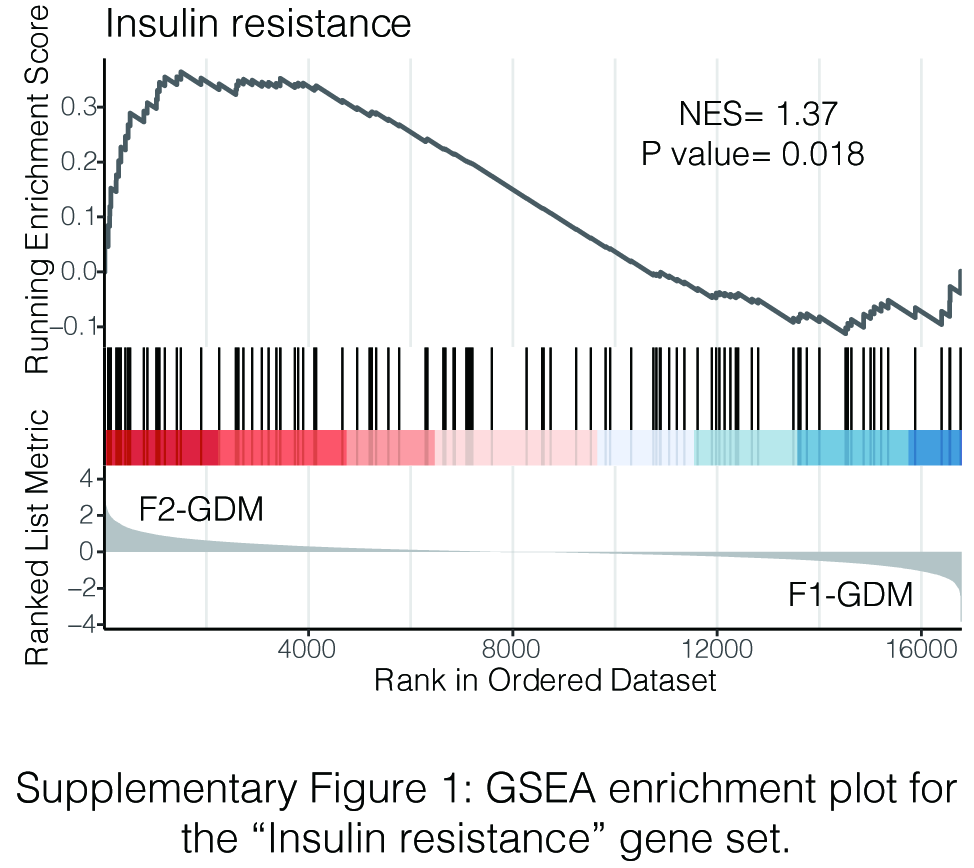

Supplement: Supplementary file 1 — Supplementary Fig 1 [file 41398_2021_1565_MOESM1_ESM.tif]

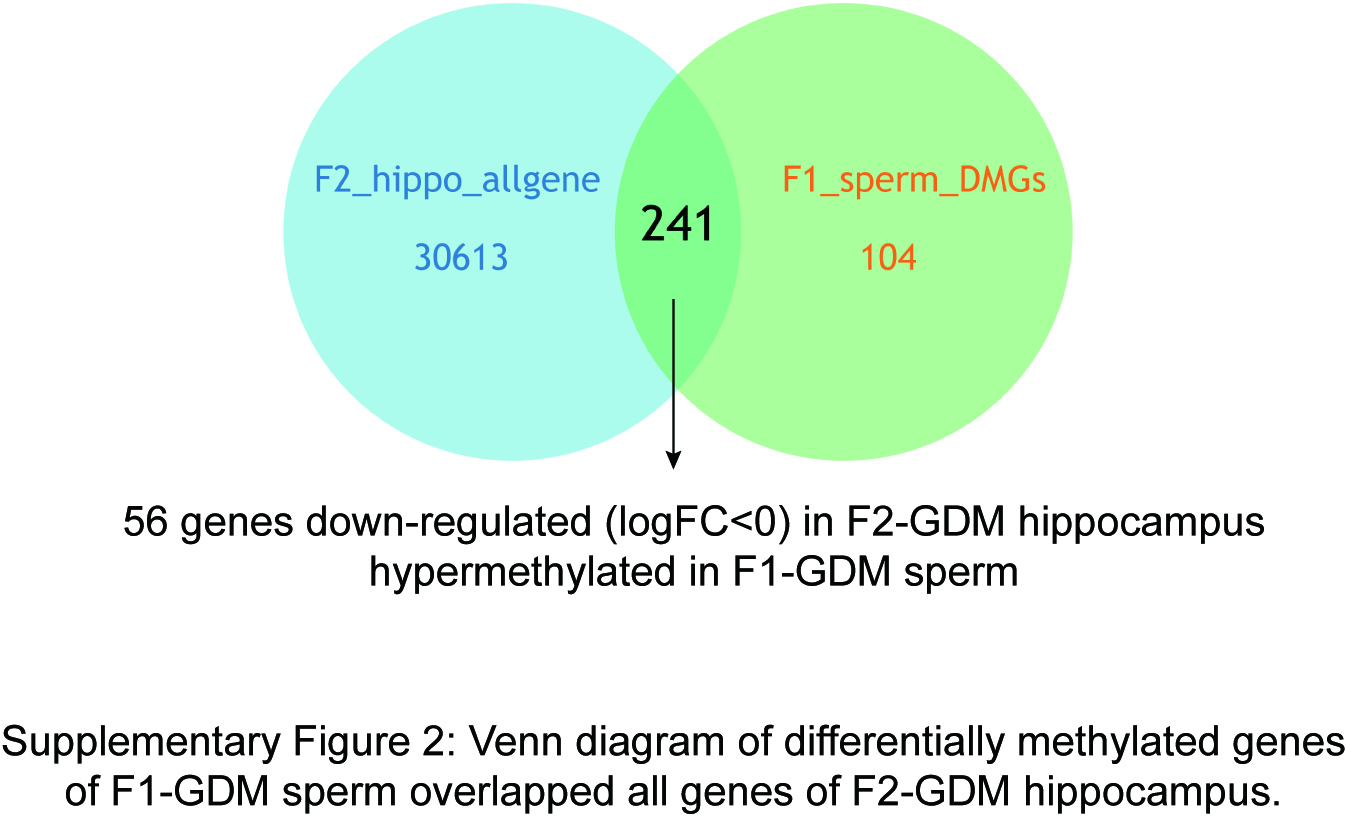

Supplement: Supplementary file 2 — Supplementary Fig 2 [file 41398_2021_1565_MOESM2_ESM.tif]
